# Constructing a consumption model of fine dining from the perspective of behavioral economics

**DOI:** 10.1371/journal.pone.0194886

**Published:** 2018-04-11

**Authors:** Sheng-Hsun Hsu, Cheng-Fu Hsiao, Sang-Bing Tsai

**Affiliations:** 1 Department of Business Administration, Chung Hua University, Hsinchu, Taiwan; 2 Ph.D. Program of Technology Management, Chung Hua University, Hsinchu, Taiwan; 3 Department of Hospitality Management, Hsing Wu University, New Taipei, Taiwan; 4 Zhongshan Institute, University of Electronic Science and Technology of China, Guangdong, China; Southwest University, CHINA

## Abstract

Numerous factors affect how people choose a fine dining restaurant, including food quality, service quality, food safety, and hedonic value. A conceptual framework for evaluating restaurant selection behavior has not yet been developed. This study surveyed 150 individuals with fine dining experience and proposed the use of mental accounting and axiomatic design to construct a consumer economic behavior model. Linear and logistic regressions were employed to determine model correlations and the probability of each factor affecting behavior. The most crucial factor was food quality, followed by service and dining motivation, particularly regarding family dining. Safe ingredients, high cooking standards, and menu innovation all increased the likelihood of consumers choosing fine dining restaurants.

## Introduction

Fine dining restaurants are operated with a high-price consumption model; to motivate consumers to pay a premium for fine dining, upscale restaurants should generate relatively high utility and satisfy highly specific needs from a behavioral economics perspective. Fine dining restaurant managers should understand consumer needs, but most managers fail to accurately comprehend and satisfy them, resulting in the withdrawal of restaurants from the market [1]. Consumers’ perceptions of fine dining restaurants have gradually shifted from exquisite traditional French cuisine and international etiquette to innovative dishes, trendy decorations, and a younger customer base [1], indicating a change in consumers’ needs. Jung and Yoon [2] believed that satisfactory restaurant services encourage revisits: consumers with variety-seeking orientation may desire to experience new things despite their satisfaction with the restaurant and thus may choose other restaurants, indicating that their hedonic motive is stronger than their benefits motive. Ponnam and Balaji [3] noted that motives may affect restaurant assessment and selection, and restaurant function must satisfy consumers’ needs to influence their decision making. Maslow [4] divided needs into five hierarchically arranged categories, namely physiological, safety, love, esteem, and self-actualization needs, which are ranked from lower-level physiological needs to higher-level psychological needs. Tikkanen [5] subjected consumers’ food service needs to hierarchical classification on the basis of Maslow’s hierarchy of needs, and published an empirical study showing that individuals have different food consumption needs. Chen, Peng, and Hung [6] stated that the reason for dining at fine dining restaurants is not merely out of basic needs; diners’ emotions and loyalties also affect the choice of fine dining restaurants. Consumers with distinct expectations display different responses toward stimuli, and their diverse demands for fine dining restaurants warrant exploration in depth.

### Consumers’ needs of fine dining restaurants

Consumers’ needs for fine dining restaurants affect restaurant selection. Using the perspective of Maslow’s hierarchy of needs to understand consumers’ needs for fine dining restaurants can inform the market positioning and policy making of fine dining restaurants. In the hierarchy of needs, physiological needs are most fundamental. Jung, Sydnor, Lee, and Almanza [7] stated that food is the main factor in choosing restaurants, with service quality and price being secondary factors. The second level of needs is safety. Röhr et al. [8] remarked that consumers have recently been concerned with food safety. Restaurants emphasize food safety because of consumers’ safety needs, and studies have noted that food safety is indeed one of the main factors in consumers’ restaurant selection [9]. The third level of needs is love. Consumers additionally exhibit other demands in restaurant selection, with their restaurant selections varying depending on their social motives; for example, the restaurants in which consumers dine with their families and friends differ from the restaurants in which they dine with their colleagues. The fourth level of needs is esteem. Voon [9] studied different types of restaurants and noted that young consumers believed that service quality is the deciding factor for choosing restaurants, although they emphasize food quality for fine dining restaurants. Consumers’ demands for service quality is a projection of their need to be respected, and Cheng et al. [10] noted that the Reliability, Responsiveness, and Assurance dimensions of the DINESERV scale, which is used to assess the service quality of fine dining restaurants, required improvements. Chin and Tsai [11] reported that customized services are essential to fine dining restaurants. Finally, self-actualization is the highest level of needs. Chen et al. [6] argued that the reason consumers choose fine dining restaurants is not only to satisfy their basic needs—it also includes differing levels of expectation. Food does not merely sustain human life: consumers emphasize both tastiness and food safety, and they choose different types of restaurants on the basis of their social needs. Apart from physical cuisine, they aspire to be respected and desire the finest service, thus, when dining becomes an art, the act of choosing fine dining restaurants satisfies consumers’ needs for esteem and self-actualization. Fine dining restaurants no longer merely provide the functions of a restaurant, namely physiological sustenance, food safety assurance, social purpose, and prestige, but they also provide psychological well-being, which is a type of self-actualization.

### Behavioral economics model for fine dining

Consumers do not have a comprehensive view of all relevant problems when they make decisions and tend to regard each factor as unique, dividing decision making into several mental accounting tasks and employing different response approaches [12,13]. Thaler [12] stated that individuals possess relevant potential concepts or behavioral representations of these mental accounting tasks, and that individual psychological factors are incorporated into behavioral decisions as explained by the mental accounting perspective. Henderson and Peterson [14] argued that mental accounting is a type of classification that enables decision makers to evaluate gains or losses on the basis of their options. Thaler [15] noted that mental accounting violates the principle of fungibility in conventional economics because different statements of mental accounting are not interchangeable. Thaler determined that decision makers assess the value of decisions according to relevant reference points, sensitivity, and loss avoidance. Assessments of gains or losses through distinct reference points yield different sensitivities, and a loss sensitivity that is high relative to gains results in loss aversion in the decision-making process. Consumers choose restaurants according to their needs; complex decision analysis processes can be simplified by the nonsubstitutability and independence of mental accounting; the classification and profit analysis of mental accounting can reduce customer sensitivity to product prices during the consumption decision-making process. A product is characterized by various attributes; the price structure of a product comprises the distinct weights of each attribute [16]. Jung et al. [7] investigated consumers’ restaurant choices using a lexicographic decision-making rule, believing that method to be suitable for analyzing psychological and behavioral economic decision making.

Research goals include confirming the factors that are key to restaurant choice, such as the effect of food, service quality, price, and dining environment [1,7,10] and exploring restaurant consumption intention under the influence of brand attitude, utility, and well-being, with well-being as the major determinant for customers’ behavioral intention [17]. Several studies have used perceived quality, customer satisfaction, overall boredom, and boredom with restaurant attributes as variety drivers and have evaluated their effect on customers’ variety-seeking intentions, with atmospheric quality, overall boredom, and boredom with atmospheric attributes having the most significant effect [18]. These studies used analysis of variance and structural equation analysis to analyze the differences and correlations between the attributes, respectively [19,20]. Despite widespread discussion on consumer behavior and selection in many studies, little cohesiveness exists in the literature on constructing consumer behavior models of fine dining restaurants from the perspective of behavioral economics. Consumer psychology is the major factor affecting the decision-making process for restaurant selection; consumers view various functions in terms of distinct and nonfungible mental accountings. Product design must consider the nonfungibility of the functions, that is, their independence. Simple and accurate models of consumer decision-making can be constructed on the basis of this concept. The decision-making process for restaurant selection is a piecemeal process that is affected by salient attributes; the determining attributes are the key factors. Studies have rarely expanded consumer needs into conceptual frameworks for the various factors of restaurant selection and have seldom analyzed the main causes that determine consumption intention. This study simulated consumer decision making for fine dining restaurants using a lexicographic decision-making rule; in that simulation, consumer needs were the consideration factor for restaurant selection. McFadden [21] illustrated utility maximizing behavior using a logistic regression model. This study used linear regression and logistic regression to model consumers’ economic behavior regarding fine dining restaurants. The purposes of this study are as follows:

a Expand consumers’ needs of fine dining restaurants into product functions using the logic of mental accounting to reduce the correlation between functions; use functions with high independence as model factors to construct a behavioral economic model for fine dining restaurants through logistic regression analysis.
b Analyze the possibility of the functions of fine dining restaurants that affect consumption intention through logistic analysis.

This study used consumers’ overall demand for fine dining restaurants to construct a theoretical framework for a behavioral economic model of fine dining restaurants. This study contributes to practical applications.

## Literature review

### Consumers’ restaurant behavioral intention

Various factors affecting restaurant selection have received scholarly attention for several decades, as shown in Table 1.

**Table 1:** Literature on factors influencing the selection of restaurants.

Scholars	Year	Factors
**Lewis [22]**	1981	Analyzed 10 factors affecting consumers’ restaurant selection involving three types of restaurants (family/popular, atmosphere, and gourmet), including food quality, menu variety, price, atmosphere, and convenience factors.
**Auty [23]**	1992	Did not subsume the factor of occasion into restaurant types, but isolated four factors (a birthday or anniversary celebration, a social occasion, a convenient/quick meal, and a business meal) from it and compiled 10 factors affecting restaurant selection from the various open-ended answers of the pilot questionnaire, namely food type, food quality, value for money, image, and atmosphere, location, speed of service, recommendations, new experiences, hours of operation, and facilities for children.
**Koo et al. [24]**	1999	Selected location, type of food, variety of food, uniqueness, car parking, price, quality, or taste of food, decoration, and service using a focus group.
**Duarte Alonso et al. [25]**	2013	Noted that among the factors affecting restaurant selection, the unique factors were healthy/nutritious food items, menu creativity, and local food use in menu development.

The aforementioned studies have indicated that consumers derived topics of health and local ingredients following economic and social development, whereas their demands on restaurants shifted from the nature of the food to food safety, cleanliness in the cooking process, and food’s social function [23], such as dining occasions and atmosphere. Recently, demand for high-quality services has increased in pursuit of self-fulfilling expectations, such as health and environmental protection, high-quality ingredients, innovation, customized demands, and high-level demands. Studies have indicated that regardless of the type of restaurant, food continues to have a high correlation with consumers’ decision-making and choice [7,22–25]. The indicators of food quality are tastiness, freshness, presentation, and menu variety. Food provides physiological satiety, but an increase in eating out also leads to increases in foodborne illness and food quality; furthermore, cooking and the cleanliness of the dining environment have also received attention, and food safety has become a concern [26]. Restaurants are locations for social communication, possess social functions, and are the agents for food innovation through the exchange of distinct food cultures [27]. They not only sell delicacies but also provide services. Stevens et al. [28] referenced the five dimensions of SERVQUAL and designed DINESERV for the catering industry, which contains the following dimensions: tangibles, reliability, responsiveness, assurance, and empathy. Chin and Tsai [11] determined that fine dining restaurant consumers attach high value to the cleanliness of the environment and equipment as well as the reliable guarantee of immediate service. Consumers not only emphasize food safety but also value meticulous service, consideration for consumers, rule flexibility for different consumers, and the capacity to satisfy consumers. Customers also want servers to make them feel distinguished, and esteem is a consumer demand that fine dining restaurants must satisfy.

Innovation may engender segmentation from peers, which is a long-term competitive advantage for restaurants [29]. Harrington et al. [1] noted that innovative characteristics are factors that influence customers’ fine dining restaurant selection. Chin and Tsai [11] considered innovation in the evaluation model they developed for fine dining restaurants, whose indicators were innovative menus, customized services, and innovative activities. Johnson et al. [30] stated that fine dining restaurants should emphasize the “taste experience in the mouth,” which is a self-actualization need; consumers can actualize themselves by experiencing the cuisine offered in fine dining restaurants.

The coding for literature analysis involves data analysis and data interpretation. Data that are classified in the same category and designated by the same code can be compared, and similar categories can be considered to constitute a theme [31–34]. After coding, factors that influence restaurant selection were categorized into the following themes: goods, services, dining motivation, dining atmosphere, dining fashion, and location. To attract customers, restaurant managers should understand consumers’ behavioral intention. Consumers not only value food quality, food safety, and service quality but are also concerned with dining motives and style.

### Behavioral economics and decision-making model

In consumers’ purchase behaviors, a rational purchase decision involves a rational measurement of the relationship between the commodity value and price, whereas an irrational purchase decision involves purchasing commodities at irrationally high prices to satisfy specific needs. Behavioral economics uses the irrational aspect of consumer behavior, that is, psychological factors, to describe their decision-making behaviors. Rick et al. [35] surveyed over 13,000 individuals, and their results indicated that most people experienced pain when losing money. Although monetary losses cause pain to people, they use money to satisfy higher-level needs. Hsee et al. [36] believed that people pursue happiness rather than money. High-price dining is not about the amount of money spent or the quantity purchased, but the amount of happiness attained. Hsee et al. [36] proposed two consumption models: in Type A consumption, consumers possess a stable scale for measuring consumption desires, making it an absolute model; in Type B consumption, consumers do not possess a stable scale for measuring consumption desires and require external reference information, making it a relative model: the purchase of luxury bags is an example of such consumption. Focusing on the consumption process rather than the expense involved results in more happiness. Dining at fine dining restaurants belongs to Type B consumption, with consumers focusing on the pleasure of consumption.

Thaler [12] proposed the concept of mental accounting, with people being affected by potential mental accounting during decision making and making decisions that differ from rational economic rules. Mental accounting is characterized by its nonfungibility, which also causes irrational decision making. The theory of mental accounting is used to explain consumers’ borrowing behaviors [37] and deferred payments [38]. Money’s utility is fixed and fungible, but individuals perform mental accounting differently during consumption, during which money’s utility is no longer fixed and fungible but possesses different values for distinct types of mental accounting. Consumers have different levels of need from fine dining restaurants; similarly, different mental accounting affects consumers’ decision making regarding fine dining restaurants; part of the money is paid to eliminate hunger and satisfy their appetites, whereas the other part is paid for food safety, dining motives, services, and dining fashion.

When consumers make decisions, they use mental accounting, and they adopt heuristic strategies for restaurant selection. Conventional decision-making studies have used strategies as hypotheses to pursue maximal benefits; conjoint analysis also attempts to obtain maximal benefits [7]. Conjoint analysis makes trade-offs between attributes in decision-making processes, which is a compensatory decision-making process [8]. Netzer et al. [39] recommended against using conjoint analysis, but recommended improvements for measuring preferences of modeled behavioral effects by combining statistics and an optimization method. Following lexicographic decision-making rules in noncompensatory decision-making models, decision makers sequentially remove nonconforming plans in accordance with the rules. Jung et al. [7] noted that people who adopt a noncompensatory decision-making model spend more money on restaurant consumption and pay more attention to food quality.

### Model construction

Satisfying consumer needs is hierarchical; restaurant fashion and atmosphere are only capable of affecting consumers’ decision making after consumers’ demands for food and service have been satisfied [3,7]. Riviere, Monrozier, Rogeaux, Pages, and Saporta [40] argued that subjecting consumer needs to level arrangement and priority processing to establish procedure-oriented product development activities establishes the ideal product. Thrane [41] noted that in hedonic price theory, a product price is a composite function of various intrinsic utility attributes. Yim et al. [16] reported that distinct products or services are combinations of various characteristics, proposing that the hedonic pricing method makes it easy to distinguish the value of characteristics, illustrating the price structure of restaurant products. Consumers’ behavioral intention may be comprehended by understanding their demand for fine dining restaurants and combining attributes that satisfy consumer needs to establish a behavioral economics model.

**Fig 1:**
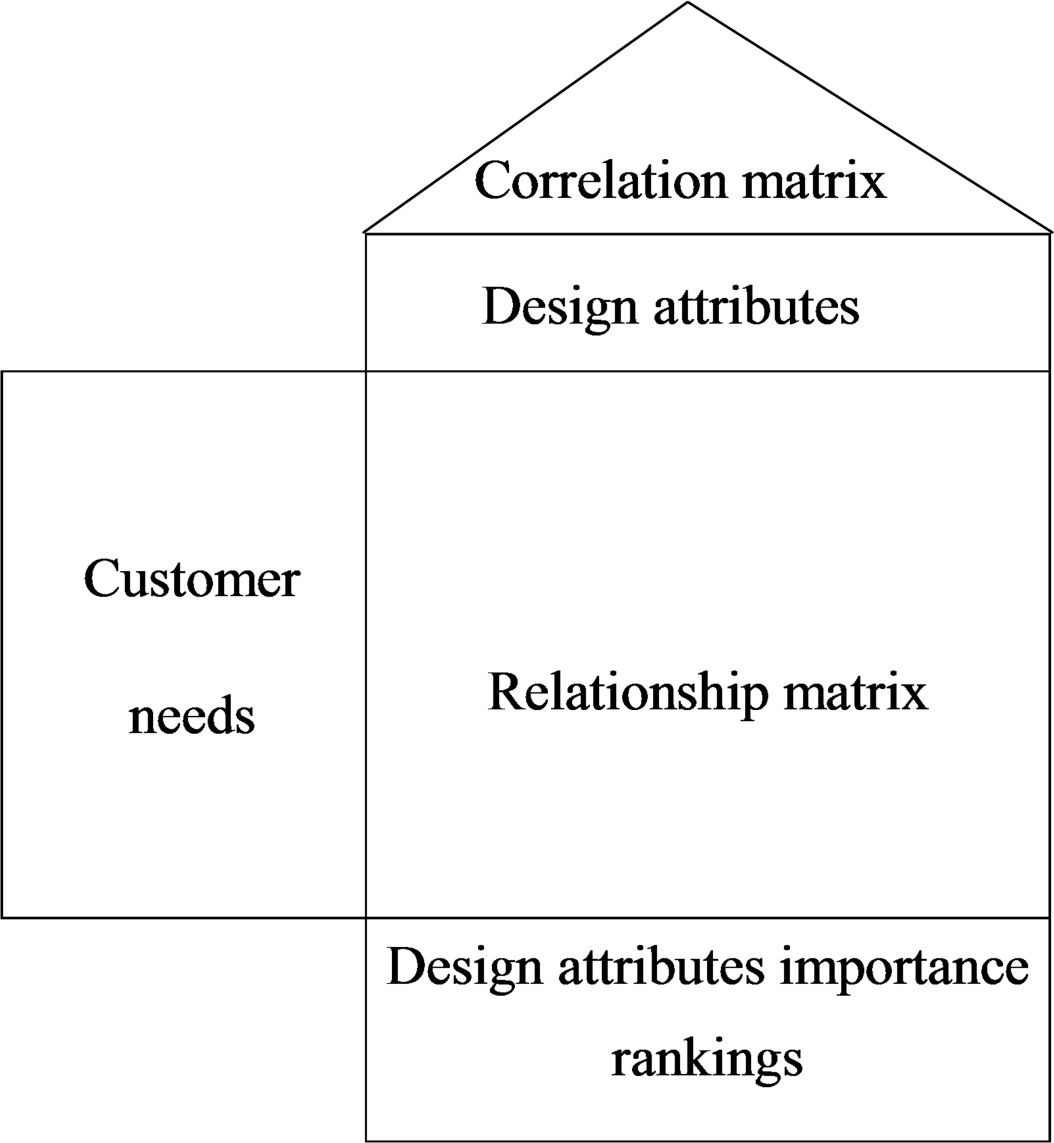
House of quality.

Quality Function Deployment (QFD) is a means of converting consumer requirements into feasible techniques to make products conform to consumer needs. Product development must proceed step by step. In accordance with the appropriate step of the relevant product development procedures, consumer needs are converted into design attributes; the relationship matrix expresses the degrees of relevance for consumer requirements and technical characteristics [42]. The relationship matrix of product quality is related to the complexity of trade-offs for design attributes, and independence between design attributes helps reduce the complexity (see Fig 1). Carnevalli et al. [43] believed that QFD faces the challenge of interpreting customers’ voices, selecting quality characteristics, and processing large matrices in its implementation; thus, they recommended an axiomatic design (AD).

Suh [44] recommended AD to satisfy the cognitive needs of products. Through consumers’ social cognition, customer needs, functional requirements (FRs), design parameters (DPs), and process variables are decomposed from high order to low order, satisfying consumer needs through reciprocal back-and-forth zigzag correspondence; a design matrix expresses the correlation between the procedural fields. Scheidl and Winkler [45] discussed AD, and opined that a conceptual model is satisfactory if it has sufficient capacity for characterization, whereas the correlation between DP and FR should be as mathematical as possible.

Consumers do not weigh several factors in the noncompensatory decision-making model. Because of the nonfungibility between factors from the perspective of mental accounting, AD is used to determine the corresponding independent DP for FR. Suh et al. [46] noted that function has unlimited demands in the face of product innovation, and the smallest possible number of FR should be adopted to satisfy customer needs [44]. Fine dining restaurant customers should also be satisfied using the smallest possible number of FR to improve customer satisfaction. The model DP in this study were based on concepts of hierarchy and independence.

## Methods

The study was reviewed and approved by an institutional review board at the Department of Technology Management, Chung Hua University (ethics committee). All participants freely decided to take part in the study or not and provided their verbal informed consent. The questionnaire was designed for a study investigating consumer behavior in fine dining restaurants, and no commercial interests are involved. No particular written consent form was necessary because the act of obtaining individual written informed consent would have compromised the anonymity of the participants’ decision to participate. Their names were not recorded, and the only personal information they were required to provide was their age and gender in order to preserve the anonymity of their responses. This consent procedure was specifically approved by the Ethics Committee mentioned above.

### Model design

**Fig 2:**
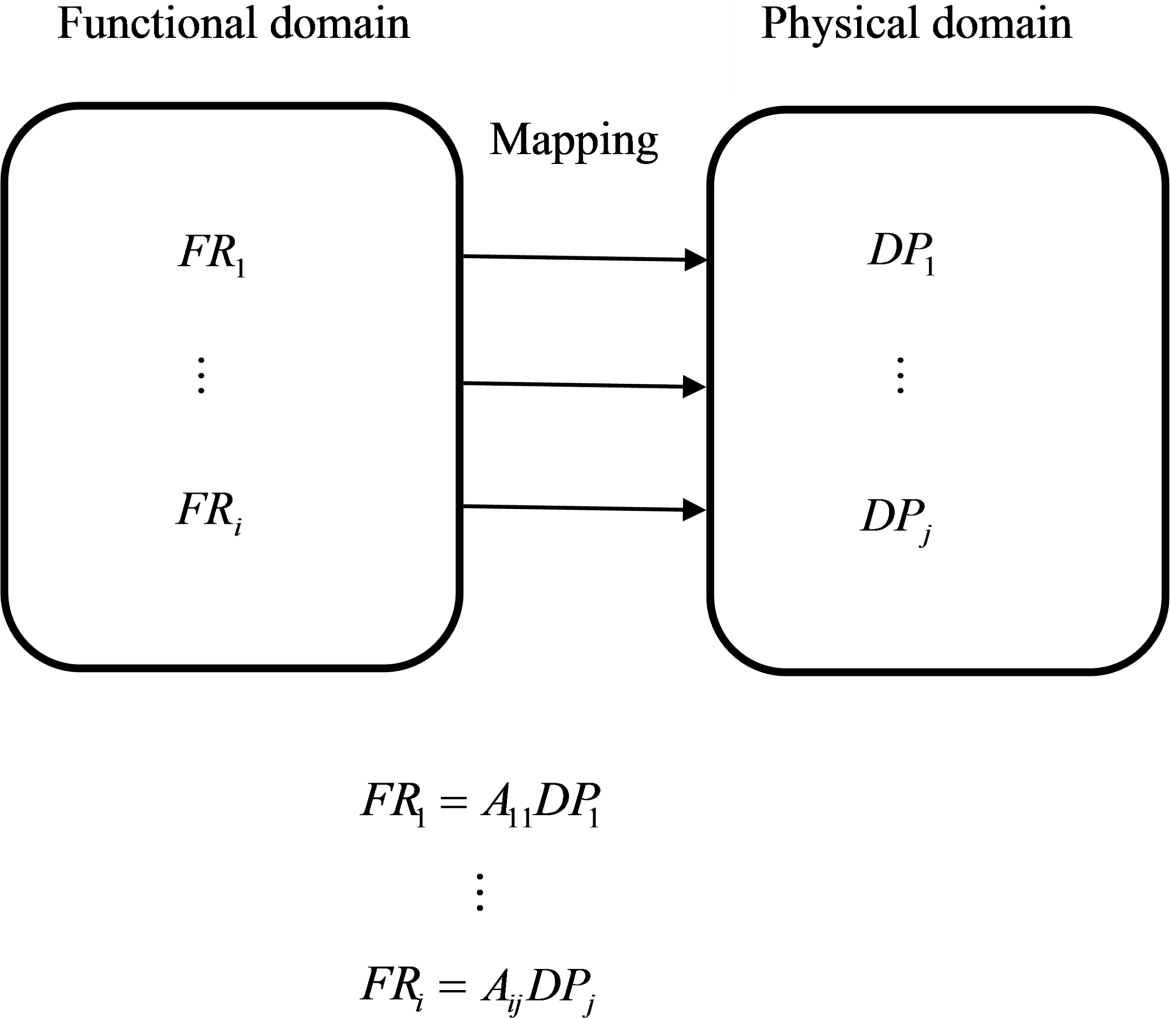
Mapping process from functional domain to physical domain.

The innovative development of a fine dining restaurant is often conducted through a sequential process from the whole to the details, and cost affects the success of a restaurant [29]. This study sequentially considered product function and used the smallest possible combination of functions to satisfy consumer needs when price was considered. The relationship matrix between the FR and DP of QFD is often complex, with a functional requirement that has multiple DP to be satisfied, whereas the parameters often have significant correlations; thus the designs are typically unsatisfactory and costly. Suh [47] proposed AD to simplify design decisions, claiming that FR and DP are independent and that the correspondence between FR and DP is one-to-one; this led to more satisfactory designs. AD is not merely a concept that optimizes designs but also a compact design that reduces costs. Consumers have different levels of needs from fine dining restaurants. Satisfying those various needs has an effect of a distinct intensity on consumers’ consumption intention. In this study, the different levels of needs were subjected to AD to map consumer needs to the FR, which were then linked one by one to the DP, that is, the influencing factors of the consumption model for fine dining restaurants, as shown in Fig 2. Among them, *A*_*ij*_ represented the correlation between functional requirements *FR*_*i*_ and DP *DP*_*j*_, with consumers being satisfied with fine dining restaurants if the FR were satisfied.

Consumers’ dining demands were compiled from the relevant literature, and the restaurant factors with low correlation and high independence were determined by seeking expert advice. Corresponding to consumers’ needs, food specifically referred to the food’s tastiness and effect on consumer satiety, whereas food safety referred to the safety of the food source and the hygiene of the cooking process. Consumer needs were converted into FR, as given in the following:

$$FR_{1}=\mathrm{physiology},\;FR_{2}=\mathrm{safety},\;FR_{3}=\mathrm{socialization},\;FR_{4}=\mathrm{esteem},\;FR_{5}=\mathrm{self-actualization}$$

The DP were inferred from the FR, as given in the following:

$$FR_{1}=A_{11}DP_{1}$$ (1)

$$FR_{2}=A_{22}DP_{2}$$ (2)

$$FR_{3}=A_{33}DP_{3}+A_{36}DP_{6}$$ (3)

$$FR_{4}=A_{44}DP_{4}$$ (4)

$$FR_{5}=A_{55}DP_{5}+A_{56}DP_{6}$$ (5)

$$DP_{1}=\mathrm{food},\;DP_{2}=\mathrm{food}\;\mathrm{safety},\;DP_{3}=\mathrm{dining}\;\mathrm{motives},\;DP_{4}=\mathrm{service},\;DP_{5}=\mathrm{dining}\;\mathrm{fashion},\;DP_{6}=\mathrm{dining}\;\mathrm{atmosphere}.$$

$$\left\{\begin{array}{c} FR_{1}\\ FR_{2}\\ FR_{3}\\ FR_{4}\\ FR_{5} \end{array}\right\}=\left[\begin{array}{cccccc} A_{11} & 0 & 0 & 0 & 0 & 0\\ 0 & A_{22} & 0 & 0 & 0 & 0\\ 0 & 0 & A_{33} & 0 & 0 & A_{36}\\ 0 & 0 & 0 & A_{44} & 0 & 0\\ 0 & 0 & 0 & 0 & A_{55} & A_{56} \end{array}\right]\left\{\begin{array}{c} DP_{1}\\ DP_{2}\\ DP_{3}\\ DP_{4}\\ DP_{5}\\ DP_{6} \end{array}\right\}$$ (6)

The factors with low correlation and high independence were determined from the aforementioned design matrix to simplify the model parameters. Dining atmosphere (*DP*_6_) was correlated with dining motives (*DP*_3_) and dining fashion (*DP*_5_), thus satisfying both socialization (*FR*_3_) and self-actualization (*FR*_5_). In an optimal design, the independence of *FRs* should be maintained. Therefore, DP were adjusted to fulfill the corresponding functional requirement of *FRs* and avoid any influence on other FRs [44]. *FR*_3_ was affected by *DP*_3_ and *DP*_6_, whereas *DP*_3_ had a higher correlation with *FR*_3_ than with *DP*_6_, with the dining motive being the satisfaction of socialization needs. Similarly, *FR*_5_ was affected by *DP*_5_ and *DP*_6_, whereas *DP*_5_ had a higher correlation with *FR*_5_ than with *DP*_6_. *DP*_3_ and *DP*_5_ had relatively low correlations with other parameters, and compared with *DP*_6_, were more able to maintain the independence of FR. Thus, *DP*_6_ can be eliminated. Simplifying the DP was recommended so that *DP*_3_ and *DP*_5_ were designed only to satisfy *FR*_3_ and *FR*_5_, respectively.

$$\left\{\begin{array}{c} FR_{1}\\ FR_{2}\\ FR_{3}\\ FR_{4}\\ FR_{5} \end{array}\right\}=\left[\begin{array}{ccccc} A_{11} & 0 & 0 & 0 & 0\\ 0 & A_{22} & 0 & 0 & 0\\ 0 & 0 & A_{33} & 0 & 0\\ 0 & 0 & 0 & A_{44} & 0\\ 0 & 0 & 0 & 0 & A_{55} \end{array}\right]\left\{\begin{array}{c} DP_{1}\\ DP_{2}\\ DP_{3}\\ DP_{4}\\ DP_{5} \end{array}\right\}$$ (7)

Among them, Aij=∂FRi∂DPj was the regression coefficient of *DP*_*j*_.

$$\left[A\right]=\left[\begin{array}{ccccc} \partial FR_{1}/\partial DP_{1} & 0 & 0 & 0 & 0\\ 0 & \partial FR_{2}/\partial DP_{2} & 0 & 0 & 0\\ 0 & 0 & \partial FR_{3}/\partial DP_{3} & 0 & 0\\ 0 & 0 & 0 & \partial FR_{4}/\partial DP_{4} & 0\\ 0 & 0 & 0 & 0 & \partial FR_{5}/\partial DP_{5} \end{array}\right]$$ (8)

The behavioral economics model for fine dining restaurant consumers was constructed through linear regression, as given in the following:

$$\begin{array}{c} \mathrm{Consumption}\;\mathrm{intention}\;=\;FR_{1}+FR_{2}+FR_{3}+FR_{4}+FR_{5}\\ =\beta_{1}\times DP_{1}+\beta_{2}\times DP_{2}+\beta_{3}\times DP_{3}+\beta_{4}\times DP_{4}+\beta_{5}\times DP_{5} \end{array}$$ (9)

### Procedures

The construction of the behavioral economics model for fine dining restaurant consumers was divided into two stages. First the effect of the DP on FR was analyzed, that is, the importance of DP [48]. Choice experiments were designed in the second stage to evaluate the possibility of DP affecting consumers’ fine dining restaurant selection. Forced choice-based experiments facilitated accurate prediction of the actual purchase decisions [7,49], and consumers’ decision making was simulated using the choice experiment.

### Research instrument

The questionnaire design referenced the Brandt [50] PRCA and the restaurant selection questionnaire by Jung et al. [8]. In this study, a 10-point Likert-type scale was used to assess the performance of each restaurant element [51,52], with the scores ranging from 1 (most unfavorable assessment) to 10 (most favorable assessment). Using a larger number of scale steps increases the confidence level of the questionnaire [53], and the percentage scale (1%–100%) was used to measure overall customer satisfaction to increase the sensitivity of satisfaction. Regarding the design of the choice experiment, the indicators of quality levels were determined according to particular studies [7]. The average performance of food involved medium levels of satiety, cooking skills, ingredient freshness, food tastiness, and value for money. The average performance of service was that the service staff had acceptably reliable professional skills, fair attitude, and solved problems in a timely manner.

A pilot study was conducted to verify the reliability of the scales; in 2017, the 30 participants were faculty members from the hospitality departments of universities in Taiwan who regularly frequented fine dining restaurants because of work requirements, and had dining experiences at fine dining restaurants within the preceding three months. The questionnaire was revised on the basis of feedback from the pilot study, and the DP were chosen. The wording was modified to improve the accuracy of the questionnaire statement and the fluency of the questionnaire.

### Data sample and collection

Chen et al. [6] noted that the price-related criteria of fine dining restaurants in Taiwan are 1) an average price of more than US$30 for the main course and 2) a set meal price (excluding service charges and tips) of more than US$67. The fine dining restaurant price of the questionnaire was established on the basis of these prices. Because this is an exploratory study, convenience sampling was performed. Consumers in fine dining restaurants who were informed of and consented to participate in the research were recruited; the targeted sample size was 150. In total, 150 participants were recruited in Northern Taiwan in 2017, all of whom had consumption experience at fine dining restaurants and consented to participate in the survey.

### Data analysis

Quantitative analysis of the questionnaire was conducted using the statistical methods of the SPSS software package, and the regression coefficient was obtained through linear regression analysis. Arora and Singer [54] noted that customer satisfaction is the main factor affecting consumption intention. The correlation *A*_*ij*_ between the DP and FR in the model was evaluated using the regression coefficient. McFadden [21] proposed a logistic regression model that accounted for decision makers’ maximized utility, a model that Jung et al. [7] employed to analyze a model for consumers’ restaurant selection behavior. The odds of the DPs affecting consumers’ fine dining restaurant selection were further analyzed using logistic regression. Overall customer satisfaction was used as the dependent variable for linear regression analysis, whereas food, food safety, dining motive, service, and dining fashion were used as the independent variables. Consumption intention was used as the dependent variable for logistic regression analysis, with 1 indicating an intention to consume and 0 indicating a lack of intention. The independent variable was the DP of the restaurant, whereas food safety, dining motivation, and food fashion were the nominal variables. Food, service, and price were distinguished depending on their attribute performance levels and designated as ordinal variables, with food and service being divided into three levels, namely inferior, average, and excellent, whereas price was divided into two levels, namely low and high.

The genders of the participants were 53.3% female and 46.7% male (Table 2). Most of the participants were aged between 20 and 39 years (60.6%). In addition, 71% of the participants had dining budgets lower than NT$400 per week; 63% of the participants spent less than NT$200 on dining out per week; 85.4% of the participants dined out more than two times a week in average.

**Table 2:** Demographic characteristics of the respondents.

Category	Response	Frequency (*N* = 192)	Percentage (%)
**Gender**	Male	70	46.7
	Female	80	53.3
**Age**	Less than 19	6	4.0
	20–29	50	33.3
	30–39	41	27.3
	40–49	34	22.7
	50–59	14	9.3
	60 or older	5	3.4
**Weekly budget for dining**	Less than $200	58	38.7
	$200–$399	49	32.7
	$400–$599	27	18.0
	$600–$799	8	5.3
	$800–$999	6	4.0
	$1,000–$1,199	1	.7
	$1,200 or more	1	.6
**Expenditure when dining out (per week)**	Less than $100	31	20.7
	$100–$199	68	45.3
	$200–$299	30	20.0
	$300–$399	10	6.7
	$400–$499	3	2.0
	$500–$599	5	3.3
	$600–$699	2	1.3
	$700 or more	1	.7
**Frequency of dining out**	Less than once per week	0	0.0
	1 or 2 times a week	22	14.6
	3 or 4 times a week	64	42.7
	5 or 6 times a week	31	20.7
	7 or 8 times a week	14	9.3
	More than 8 times a week	19	12.7

## Results

The regression analysis was first conducted using gender and age as control variables. The result showed that the influences of gender and age on the choice of fine dining restaurants were nonsignificant (Table 3).

**Table 3:** Importance and satisfaction of fine dining restaurant functions.

Rank	Attributes	Regression coefficients	Sig.
**1**	Food	.272**	.002
**2**	Food safety	.045	.595
**3**	Dining motivation	.207**	.006
**4**	Service	.226**	.006
**5**	Dining fashion	.095	.238
**6**	Gender	-.027	.703
**7**	Age	-.003	.969

*R*^2^ = .374.

**P < .01.

Because of the nonsignificant influence of gender and age, they were excluded from the model construction. The Pearson’s correlation coefficients for the attributes were between 0.216 and 0.550, indicating that the attributes were not highly correlated with each other, as shown in Table 4.

**Table 4:** Correlation matrix of fine dining restaurant attributes.

Item	Attributes	Mean	S.D.	Item 1	Item 2	Item 3	Item 4	Item 5
**1**	Food	8.2600	.97230	1	.550**	.375**	.391**	.382**
**2**	Food safety	8.3667	1.02595		1	.286**	.459**	.368**
**3**	Dining motivation	7.7400	1.40196			1	.330**	.216**
**4**	Service	8.5467	1.28275				1	.434**
**5**	Dining fashion	8.3000	1.23566					1

**P < .01.

### Reliability and validity analysis

Reliability and validity were assessed with confirmation factor analysis. This study adopted Cronbach’s α to verify the reliability of the questionnaire samples. The result indicated that the Cronbach’s α value was 0.738, which was above the benchmark of 0.70 suggested by Nunnally [55]. This result indicated high construct reliability, and thus the reliability of the scales was ensured.

Convergence validity was analyzed using the maximum likelihood method. The factor loads of questionnaire items were used as the criteria for convergence validity. The analysis results displayed in Table 5 showed that the factor loads of the items ranged between 0.552 and 0.721 at the 0.05 level of significance, indicating a satisfactory convergence validity of the questionnaire (Hair et al., 2006)[56].

**Table 5:** Importance and satisfaction of fine dining restaurant functions.

Rank	Attributes	Factor loading	Regression coefficients	Mean	S.D.
**1**	Food	.716	.275**	8.2600	.97230
**2**	Food safety	.721	.045	8.3667	1.02595
**3**	Dining motivation	.463	.206**	7.7400	1.40196
**4**	Service	.633	.225**	8.5467	1.28275
**5**	Dining fashion	.552	.087	8.3000	1.23566

*R*^2^ = .352.

**P < .01.

### Behavioral economics model for fine dining

The importance of restaurant functions for satisfying consumers’ needs was analyzed to construct the behavioral economics model for fine dining restaurants.

Importance was determined through regression analysis, as shown in Table 1. The Durbin–Watson value of 1.704, which is between 1.5 and 2.5, indicated no autocorrelation problem in the model. All variance inflation factor values were lower than 10, which indicated that multicollinearity was statistically nonsignificant. The behavioral economics model was estimated through regression coefficients, as given in the following:

$$\begin{array}{l} FR_{1}+FR_{2}+FR_{3}+FR_{4}+FR_{5}\\ =0.275\times DP_{1}+0.045\times DP_{2}+0.206\times DP_{3}+0.225\times DP_{4}+0.087\times DP_{5} \end{array}$$ (10)

The results indicated that food, dining motivation, and service significantly affected consumers’ behavioral intentions regarding fine dining restaurants (Table 5). Food was the key factor in satisfying consumer needs, whereas food safety and dining fashion did not significantly influence customer satisfaction. High service performance considerably satisfied consumers, whereas satisfaction levels derived from food and dining motivation were lower.

### Consumer fine dining restaurant selection

The likelihood of consumers choosing fine dining restaurants was investigated through logistic regression. Table 6 shows the odds ratios of food and service affecting consumer selection. The omnibus tests of model coefficients possessed low significance (0.000). The model diagnostic was adequate at the Nagelkerke value of 0.497 [57]. The odds ratio of service was 5.678, whereas that of food affecting restaurant selection was 7.861, indicating that food affects selection more significantly than does service.

**Table 6:** Odds ratios of food and service affecting consumer selection.

Design parameters	Logistic regression			
	Regression coefficients	S.D.	Odds ratio	95% *CI*
**Food quality**	2.062**	.123	7.861	[6.178, 10.001]
**Service quality**	1.737**	.112	5.678	[4.558, 7.073]

CI = confidence interval.

**P < 0.01.

For food and service, high and low performance affected consumer restaurant selection with variation in likelihood (Table 7). The food and service quality were ordinal variables that were divided into poor, average, and good levels, and the average level was used as the reference group (i.e., reference level). The omnibus tests of model coefficients revealed low significance (0.000). For consumer fine dining restaurant selection, low performance in food and service adversely affected selection. Low food and service performance had a greater effect on consumer restaurant selection than did high performance.

**Table 7:** Selection experiment results for food and service quality levels.

Design parameters	Logistic regression			
	Regression coefficients	S.D.	Odds ratio	95% *CI*
**Food quality (reference level: average)**				
Good service	.978**	.211	2.660	[1.760, 4.019]
Poor service	-3.053**	.258	.047	[.028, .078]
**Service quality (reference level: average)**				
Good service	.672**	.225	1.957	[1.259, 3.043]
Poor service	-2.484**	.223	.083	[.054, .129]

*CI* = confidence interval.

**P < 0.01.

Variation in the food safety attributes affecting restaurant selection is shown in Table 8. Food safety is a nominal variable, involving hypoallergenic food, relatively safe food ingredients, and cooking standards; hypoallergenic food was used as the reference group. The omnibus tests of model coefficients indicated low significance (0.000). Hypoallergenic food was the reference level for examining how different food safety attributes influenced consumer selection. The odds ratios of safe food ingredients and cooking standards were 2.992 and 2.961, respectively. In other words, a restaurant with notably safe ingredients or cooking standards had a 2.9 times higher likelihood of being selected over a similar restaurant with hypoallergenic food.

**Table 8:** Selection experiment results for food safety.

Design parameters	Logistic regression			
	Regression coefficients	S.D.	Odds ratio	95% *CI*
**Hypoallergenic food (reference factor)**				
Safe food ingredient	1.096**	.140	2.992	[2.274, 3.936]
Cooking standard	1.085**	.140	2.961	[2.251, 3.894]

*CI* = confidence interval.

**P < 0.01.

Table 9 shows variation in the dining motive attributes affecting restaurant selection. Dining motivation is a nominal variable and was divided into groups of dine with group of friends and associates, dine with family, and dine with intimate acquaintances; dine with group of friends and associates was used as the reference group. The omnibus tests of model coefficients revealed a low significance value of .000. The odds of choosing a restaurant to dine with family over a restaurant to dine with a group of friends and associates was 3.019 to 1, whereas the odds of choosing a restaurant to dine with intimate acquaintances had odds of 1.856 to 1 over those of choosing a restaurant to dine with a group of friends and associates. In other words, the odds of choosing a restaurant for dining with family were 1.627 times higher than the chance of choosing a restaurant for dining with intimate acquaintances.

**Table 9:** Selection experiment results for dining motivation.

Design parameters	Logistic regression			
	Regression coefficients	S.D.	Odds ratio	95% *CI*
**Dine with a group of friends and associates (reference factor)**				
Dine with family	1.105**	.139	3.019	[2.298, 3.967]
Dine with intimate ones	.618**	.135	1.856	[1.425, 2.418]

*CI* = confidence interval.

**P < 0.01.

Regarding dining fashion, variation in the attributes affecting restaurant selection is shown in Table 10. Dining fashion was a nominal variable, divided into top ingredients, media recommendation, and menu innovation; top ingredients served as the reference group. The omnibus tests of model coefficients revealed low significance (0.000). A restaurant that possessed media recommendations had 2.008 times lower odds of being selected over a similar restaurant that featured top ingredients. A restaurant that featured menu innovation had 2.496 times higher odds of being selected over a similar restaurant that possessed media recommendations.

**Table 10:** Selection experiment results for dining fashion.

Design parameters	Logistic regression			
	Regression coefficients	S.D.	Odds ratio	95% *CI*
Top ingredients (reference factor)				
Media recommendation	-.697**	.136	.498	[.382, .650]
Menu innovation	.218	.135	1.243	[.954, 1.619]

*CI* = confidence interval.

**P < 0.01.

In this study, the key functions of fine dining restaurants were food, service, and dining motivation. The results suggested that food and service performance should be maintained to meet consumer requirements. In particular, food performance should be prioritized. The results also showed that when consumers choose fine dining restaurants, dining with family is more influential than other motivations. These data imply that safe food ingredients, high cooking standards, and menu innovation are attractive attributes in fine dining restaurants that may increase the likelihood of consumers selecting particular restaurants.

## Discussion

### Research findings

In this study, mental accounting was used to investigate consumers’ behavioral economics model for fine dining restaurants. This study determined that consumers’ key FR for fine dining restaurants echoed Maslow’s [4] hierarchy of needs. Consumers performed independent mental accounting for each level of needs, and the FR were expanded into DP through QFD, after which the model design was simplified using AD [43]. Consumers’ behavioral economics model for fine dining restaurants was constructed through two-stage analysis. The results indicated that the key functions affecting consumers’ fine dining restaurant selection, in the order of importance, were food, service, dining motivation, food safety, and dining fashion. Thus, consumers’ attention to food and service was consistent with particular studies [7,58].

This study also found that dining motivation was a factor influencing the selection of restaurants. Consumers who dine with family and choose fine dining restaurants are the target market restaurateurs should pay attention to. Safe food ingredients and cooking standards (in the attribute of food safety) and menu innovation (in the attribute of dining fashion) can also increase consumers’ intention to dine at fine dining restaurants. The construction of a consumer behavior model that reflects consumers’ needs enables restaurant owners to know individual factors that influence restaurant selection, to comprehensively understand consumers’ needs, and to help managers to position and to manage fine dining restaurants in the target market. Improving food performance may increase consumers’ consumption intention. However, the effects of dining fashion and food safety were nonsignificant, which may mean that consumers are reassured of the food safety at fine dining restaurants. In terms of cognition, individuals select fine dining restaurants to publicize themselves and demonstrate a unique dining fashion; however, the results were nonsignificant. Food is a basic physiological requirement; it has a substantial effect on customer satisfaction and is also the principal factor that affects consumers’ restaurant selection. Notably, the present study determined that both dining motivation and service are crucial for fine dining restaurant. The possibility that attribute performances and DP affect consumers’ fine dining restaurant selection was further investigated using a choice experiment. In terms of social needs, the possibility that dining with family affects restaurant selection was higher than for other dining motivation, with consumers being very willing to pay high prices to dine with family; thus, fine dining restaurants that appeal to those who aspire to dine with their families may be favored by large majorities of the dining public. Improving food performance seemed to yield a greater consumer utility than did enhancing service performance, hence food quality increased the likelihood that customers would choose fine dining restaurants. In terms of food safety, consumers focused on choosing restaurants whose cooking standards were high rather than those using safe food ingredients. They may trust the ingredient selection process of fine dining restaurants but retain doubts about their cooking processes. Recent advances in the Internet have made consumers more skilled at obtaining culinary knowledge than they previously had been, thus the Internet has reduced the effect of media recommendations [59]. Among various dining fashions, menu innovation is the most likely to attract consumers; restaurants that provide unique dish innovations and conduct market segmentation are the most likely to succeed.

### Theoretical and practical implications

Matzler et al. [48] divided product attributes into three factors: the basic factor, excitement factor, and performance factor. The basic factor influences customer dissatisfaction, the excitement factor affects customer satisfaction, and the performance factor affects both. Even if the excitement factor and performance factor exhibit high performance, customers tend to remain dissatisfied if the basic factor does not meet their demands [60]. Jung et al. [7] stated that service affects consumer dissatisfaction, and even a high performance fails to increase consumer satisfaction, thus making it a basic factor. However, a high food performance yields a high consumer satisfaction, and vice versa; thus, it is a performance factor. Although fine dining restaurants often possess high-quality service, luxury, top ingredients, and innovation, these factors are marketing appeals and contribute to consumers’ higher-level needs of esteem and self-actualization. High-level needs are excitement factors, although consumers may be dissatisfied if fine dining restaurants are unable to provide food and service that satisfies them. Food is the primary component of the purchase, and consumers tend to exhibit loss aversion regarding its performance in that most consumers avoid restaurants with low-quality service, low prices, and low-quality food [7,58].

The obtained utility is the result of comparison between consumers’ perception of a commodity’s value and the actual amount of money to be paid [12]. Zeithaml [61] stated that consumers determine the perceived value on the basis of an overall assessment of the amount paid for a product and the utility obtained. Kahneman and Tversky [62] proposed the prospect theory and explored decision-making behaviors through human psychology, arguing that value is determined by changes in wealth instead of by the wealth level of the expected utility theory. For consumers, the evaluation of mental accounting depends on options (differences in values) rather than the value of the difference [15]. Wiedmann et al. [63] established the luxury value for luxury goods using financial value, functional value, personal value, and social value, with luxury value affecting consumption behavior. Consumers possess a conservative evaluation attitude for food costs and more intolerant toward failure of food function value to satisfy needs. The mental accounting of food function is affected by consumer psychology, and loss sensitivity is greater than gain sensitivity, according to the value function of prospect theory [62]. Thaler [15] noted that mental accounting affects consumers’ decision making. Thaler [16] proposed hedonic editing, where people prefer to subject gain and loss to integration and segregation, respectively, to maximize values. Decision makers construct a model of how consumers evaluate events through mental accounting arithmetic [15]. Considering the mental accounting of various factors, managers should first allocate resources to food, and then invest resources into satisfying dining motivation and service after the food meets consumers’ demands. Food safety should then be enhanced if extra capacity remains, and unique dining fashions should be used for segmentation competition.

The behavioral economics model for fine dining restaurants established in this study helps explain consumers’ assessment of the restaurant function, which also affects consumption intention. The proposed model delivers a detailed analysis of the possibility of food and service performance levels affecting consumption intention, and clearly reveals the types of food safety, dining motivation, and dining fashion demands that most impress consumers. This study also verified that food is still the competitive advantage for fine dining restaurants. In terms of practical applications, apart from luxury, fine dining restaurant marketing should not neglect the fact that consumers attach the highest value to food. Consumers are willing to pay a high price to obtain the utility of food, thus fine dining restaurant managers should not sacrifice food quality performance for luxurious decoration [64]. Among consumers’ psychological characteristics, Lee and Hwang [65] reported that materialism and hedonism hold favorable attitudes toward fine dining restaurants, whereas consumers’ uniqueness holds unfavorable attitudes. Both physiological and psychological needs should be satisfied, and dining with family results in a sense of happiness; thus, marketing should appeal to restaurants that are suitable for dining with family. Restaurants are highly substitutable in fierce market competition scenarios, and consumers are likely to be dissatisfied when restaurants do not meet their expected performance. Using the proposed model in this study, managers may more thoroughly understand consumers’ FR for fine dining restaurants, while enhancing material comforts and satisfying consumers’ materialistic needs through food and food safety. The winning strategy is to provide a dining experience that is worthwhile because it satisfies dining motivation, service, and dining fashion to satisfy consumers’ hedonic needs. Theoretically, this study compiled the factors scattered in various consumer behavior studies on restaurants to establish an overall framework. Practically, the model proposed in this study was straightforward to use; this is expected to facilitate its use by restaurant managers. The market is highly changeable; through the model framework, managers can fully understand consumers’ needs and the deficiencies in restaurants’ performance, thereby formulating more attractive market positioning and branding strategies for fine dining restaurants.

### Limitations and future studies

Although the behavioral economics model constructed in this study was simple and easy to use, it also contained several limitations. First, because of the limited participant sample, the inference of the model was limited to populations with similar characteristics. Second, to simplify the model, not all potential factors were included: only the crucial factors were selected, whereas some factors were deleted.

This study used fine dining restaurants as the research subject, and consumers’ key demands for fine dining restaurants may be comprehended if future studies use other restaurants as their study subject for comparison. In terms of depth, the DP should be expanded under the five major functions to accurately express consumer needs. In terms of breadth, the behavioral economics model constructed may be applied to other types of restaurants to analyze the differences in consumers’ perception and demand for fine dining restaurants and other restaurants.

## Supporting information

S1 File Study questionnaire in English. (DOCX)

S2 File Study questionnaire in Chinese. (DOCX)
