# Supplementary material for: Constructing a consumption model of fine dining from the perspective of behavioral economics
Source: PLoS One. 2018 Apr 11;13(4):e0194886. doi: 10.1371/journal.pone.0194886 (PMC5895011; doi:10.1371/journal.pone.0194886)
Supplement: S1 File — (DOCX) [file pone.0194886.s001.docx]

**Questionnaire of Consumer Behavior in Fine Dining Restaurants**

| Dear consumers:  This is a questionnaire designed for a study investigating consumer behavior in fine dining restaurants. We sincerely invite you to fill this questionnaire in order to understand factors affecting your selection of fine dining restaurants. This survey is anonymous, and the results would only be used for academic purposes; no commercial interests are involved. We highly appreciate your participation in this research.  Ph.D. Program of Technology Management, Chung Hua University  Sheng-Hsun Hsu  Cheng-Fu Hsiao |
| --- |

1. Demographic Information

1. Gender: □Male □Female

2. Age □younger than 20 □20–29 □30–39 □40–49 □50–59 □60 years old or older

3. How much is your dining budget per week (in NT dollars)?

□less than 6,000 □6,000–11,999 □12,000–17,999 □18,000–23,999
□24,000–29,999 □30,000–35,999 □36,000–41,999 □42,000 or more

4. How much money do you spend averagely on dining out per week (in NT dollars)?
□less than 3,000 □3,000–5,999 □6,000–8,999 □9,000–11,999
□12,000–14,999 □15,000–17,999 □18,000–20,999 □21,000 or more

5. How many times do you dine out per week on average?
□0 □1–2 □3–4 □5–6 □7–8 □8 times or more

1. Performance of Quality Attributes of Fine Dining Restaurants and Overall Satisfaction

If you have visited a fine dining restaurant, please indicate your level of satisfaction with the following five quality attributes. Please also give a score for your overall level of satisfaction with this restaurant according to the performance of the five attributes.

| **Quality attributes of a restaurant** | **Very unsatisfied, Unsatisfied, Slightly unsatisfied, Slightly satisfied, Satisfied, Very satisfied** | | | | | | | | | |
| --- | --- | --- | --- | --- | --- | --- | --- | --- | --- | --- |
|  | 1 | 2 | 3 | 4 | 5 | 6 | 7 | 8 | 9 | 10 |
| 1. Dishes and taste  (Food) | □ | □ | □ | □ | □ | □ | □ | □ | □ | □ |
| 2. Food ingredient safety and hygiene during the cooking process  (Food safety) | □ | □ | □ | □ | □ | □ | □ | □ | □ | □ |
| 3. Fulfillment of your socialization needs  (Dining motivation) | □ | □ | □ | □ | □ | □ | □ | □ | □ | □ |
| 4. Services provided by the restaurant  (Service) | □ | □ | □ | □ | □ | □ | □ | □ | □ | □ |
| 5. Dining fashion and characteristics of the restaurant  (Dining fashion) | □ | □ | □ | □ | □ | □ | □ | □ | □ | □ |
| * Please give a score (0–100 points) for your satisfaction with the overall performance of this fine dining restaurant: _______. | | | | | | | | | | |

1. Situational Description

In a holiday evening, you are going to dine out due to certain reasons. Two fine dining restaurants at similar locations and with similar transportation convenience are available for you to choose. Factors of food, food safety, dining motivation, service, and dining fashion may influence your choice of the restaurant.

In Section Ⅳ, you have different considerations regarding food safety, dining motivation, and dining fashion, and the two restaurants have dissimilar levels of food and service performance and are different in price. Please select a restaurant you prefer according to your consideration of food, food safety, dining motivation, service, and dining fashion.

Please refer to the descriptions of restaurants’ average performance in certain attributes.

1. Average level of food: Medium levels of satiety, cooking skills, ingredient freshness, food tastiness, and value for money

2. Average level of service: The service staff had acceptably reliable professional skills, fair attitude, and solved problems in a timely manner

3. Acceptable price: The price is acceptable for a fine dining restaurant

1. Restaurant Choice
2. Two restaurants A and B satisfy all your needs except for the attributes of food safety and price. Please choose a restaurant you prefer to visit.

| Attributes | Restaurant A | Restaurant B |
| --- | --- | --- |
| Food safety | Unsatisfied with the safety of food ingredient | Satisfied with the safety of food ingredient |
| Price | Low | High |
| Which restaurant would you choose? | □Restaurant A | □Restaurant B |

1. Two restaurants A and B satisfy all your needs except for the attributes of food safety and price. Please choose a restaurant you prefer to visit.

| Attributes | Restaurant A | Restaurant B |
| --- | --- | --- |
| Food safety | Satisfied with the hygiene during the cooking process | Satisfied with the safety of food ingredient |
| Price | Low | High |
| Which restaurant would you choose? | □Restaurant A | □Restaurant B |

1. Two restaurants A and B satisfy all your needs except for the attributes of food safety and price. Please choose a restaurant you prefer to visit.

| Attributes | Restaurant A | Restaurant B |
| --- | --- | --- |
| Food safety | Hypoallergenic food | Satisfied with the safety of food ingredient |
| Price | Low | High |
| Which restaurant would you choose? | □Restaurant A | □Restaurant B |

1. Two restaurants A and B satisfy all your needs except for the attributes of food safety and price. Please choose a restaurant you prefer to visit.

| Attributes | Restaurant A | Restaurant B |
| --- | --- | --- |
| Food safety | Unsatisfied with the safety of food ingredient | Satisfied with the hygiene during the cooking process |
| Price | Low | High |
| Which restaurant would you choose? | □Restaurant A | □Restaurant B |

1. Two restaurants A and B satisfy all your needs except for the attributes of food safety and price. Please choose a restaurant you prefer to visit.

| Attributes | Restaurant A | Restaurant B |
| --- | --- | --- |
| Food safety | Satisfied with the safety of food ingredient | Satisfied with the hygiene during the cooking process |
| Price | Low | High |
| Which restaurant would you choose? | □Restaurant A | □Restaurant B |

1. Two restaurants A and B satisfy all your needs except for the attributes of food safety and price. Please choose a restaurant you prefer to visit.

| Attributes | Restaurant A | Restaurant B |
| --- | --- | --- |
| Food safety | Hypoallergenic food | Satisfied with the hygiene during the cooking process |
| Price | Low | High |
| Which restaurant would you choose? | □Restaurant A | □Restaurant B |

1. Two restaurants A and B satisfy all your needs except for the attributes of food safety and price. Please choose a restaurant you prefer to visit.

| Attributes | Restaurant A | Restaurant B |
| --- | --- | --- |
| Food safety | Allergic food | Hypoallergenic food |
| Price | Low | High |
| Which restaurant would you choose? | □Restaurant A | □Restaurant B |

1. Two restaurants A and B satisfy all your needs except for the attributes of food safety and price. Please choose a restaurant you prefer to visit.

| Attributes | Restaurant A | Restaurant B |
| --- | --- | --- |
| Food safety | Satisfied with the safety of food ingredient | Hypoallergenic food |
| Price | Low | High |
| Which restaurant would you choose? | □Restaurant A | □Restaurant B |

1. Two restaurants A and B satisfy all your needs except for the attributes of food safety and price. Please choose a restaurant you prefer to visit.

| Attributes | Restaurant A | Restaurant B |
| --- | --- | --- |
| Food safety | Satisfied with the hygiene during the cooking process | Hypoallergenic food |
| Price | Low | High |
| Which restaurant would you choose? | □Restaurant A | □Restaurant B |

1. Two restaurants A and B satisfy all your needs except for the attributes of food safety and price. Please choose a restaurant you prefer to visit.

| Attributes | Restaurant A | Restaurant B |
| --- | --- | --- |
| Food safety | Meet the requirements of food safety | Do not meet the requirements of food safety |
| Price | Low | High |
| Which restaurant would you choose? | □Restaurant A | □Restaurant B |

1. Two restaurants A and B satisfy all your needs except for the attributes of dining motivation and price. Please choose a restaurant you prefer to visit.

| Attributes | Restaurant A | Restaurant B |
| --- | --- | --- |
| Dining motivation | Be unsuitable for dining with family | Be suitable for dining with family |
| Price | Low | High |
| Which restaurant would you choose? | □Restaurant A | □Restaurant B |

1. Two restaurants A and B satisfy all your needs except for the attributes of dining motivation and price. Please choose a restaurant you prefer to visit.

| Attributes | Restaurant A | Restaurant B |
| --- | --- | --- |
| Dining motivation | Be suitable for dining with intimate ones | Be suitable for dining with family |
| Price | Low | High |
| Which restaurant would you choose? | □Restaurant A | □Restaurant B |

1. Two restaurants A and B satisfy all your needs except for the attributes of dining motivation and price. Please choose a restaurant you prefer to visit.

| Attributes | Restaurant A | Restaurant B |
| --- | --- | --- |
| Dining motivation | Be suitable for dining with a group of friends and associates | Be suitable for dining with family |
| Price | Low | High |
| Which restaurant would you choose? | □Restaurant A | □Restaurant B |

1. Two restaurants A and B satisfy all your needs except for the attributes of dining motivation and price. Please choose a restaurant you prefer to visit.

| Attributes | Restaurant A | Restaurant B |
| --- | --- | --- |
| Dining motivation | Be unsuitable for dining with intimate ones | Be suitable for dining with intimate ones |
| Price | Low | High |
| Which restaurant would you choose? | □Restaurant A | □Restaurant B |

1. Two restaurants A and B satisfy all your needs except for the attributes of dining motivation and price. Please choose a restaurant you prefer to visit.

| Attributes | Restaurant A | Restaurant B |
| --- | --- | --- |
| Dining motivation | Be unsuitable for dining with family | Be suitable for dining with intimate ones |
| Price | Low | High |
| Which restaurant would you choose? | □Restaurant A | □Restaurant B |

1. Two restaurants A and B satisfy all your needs except for the attributes of dining motivation and price. Please choose a restaurant you prefer to visit.

| Attributes | Restaurant A | Restaurant B |
| --- | --- | --- |
| Dining motivation | Be suitable for dining with a group of friends and associates | Be suitable for dining with intimate ones |
| Price | Low | High |
| Which restaurant would you choose? | □Restaurant A | □Restaurant B |

1. Two restaurants A and B satisfy all your needs except for the attributes of dining motivation and price. Please choose a restaurant you prefer to visit.

| Attributes | Restaurant A | Restaurant B |
| --- | --- | --- |
| Dining motivation | Be unsuitable for dining with a group of friends and associates | Be suitable for dining with a group of friends and associates |
| Price | Low | High |
| Which restaurant would you choose? | □Restaurant A | □Restaurant B |

1. Two restaurants A and B satisfy all your needs except for the attributes of dining motivation and price. Please choose a restaurant you prefer to visit.

| Attributes | Restaurant A | Restaurant B |
| --- | --- | --- |
| Dining motivation | Be unsuitable for dining with family | Be suitable for dining with a group of friends and associates |
| Price | Low | High |
| Which restaurant would you choose? | □Restaurant A | □Restaurant B |

1. Two restaurants A and B satisfy all your needs except for the attributes of dining motivation and price. Please choose a restaurant you prefer to visit.

| Attributes | Restaurant A | Restaurant B |
| --- | --- | --- |
| Dining motivation | Be unsuitable for dining with intimate ones | Be suitable for dining with a group of friends and associates |
| Price | Low | High |
| Which restaurant would you choose? | □Restaurant A | □Restaurant B |

1. Two restaurants A and B satisfy all your needs except for the attributes of dining motivation and price. Please choose a restaurant you prefer to visit.

| Attributes | Restaurant A | Restaurant B |
| --- | --- | --- |
| Dining motivation | Meet the dining motive | Do not meet the dining motive |
| Price | Low | High |
| Which restaurant would you choose? | □Restaurant A | □Restaurant B |

1. Two restaurants A and B satisfy all your needs except for the attributes of dining fashion and price. Please choose a restaurant you prefer to visit.

| Attributes | Restaurant A | Restaurant B |
| --- | --- | --- |
| Dining fashion | No media recommendation | Media recommendation |
| Price | Low | High |
| Which restaurant would you choose? | □Restaurant A | □Restaurant B |

1. Two restaurants A and B satisfy all your needs except for the attributes of dining fashion and price. Please choose a restaurant you prefer to visit.

| Attributes | Restaurant A | Restaurant B |
| --- | --- | --- |
| Dining fashion | Menu innovation | Media recommendation |
| Price | Low | High |
| Which restaurant would you choose? | □Restaurant A | □Restaurant B |

1. Two restaurants A and B satisfy all your needs except for the attributes of dining fashion and price. Please choose a restaurant you prefer to visit.

| Attributes | Restaurant A | Restaurant B |
| --- | --- | --- |
| Dining fashion | Top ingredients | Media recommendation |
| Price | Low | High |
| Which restaurant would you choose? | □Restaurant A | □Restaurant B |

1. Two restaurants A and B satisfy all your needs except for the attributes of dining fashion and price. Please choose a restaurant you prefer to visit.

| Attributes | Restaurant A | Restaurant B |
| --- | --- | --- |
| Dining fashion | No menu innovation | Menu innovation |
| Price | Low | High |
| Which restaurant would you choose? | □Restaurant A | □Restaurant B |

1. Two restaurants A and B satisfy all your needs except for the attributes of dining fashion and price. Please choose a restaurant you prefer to visit.

| Attributes | Restaurant A | Restaurant B |
| --- | --- | --- |
| Dining fashion | Media recommendation | Menu innovation |
| Price | Low | High |
| Which restaurant would you choose? | □Restaurant A | □Restaurant B |

1. Two restaurants A and B satisfy all your needs except for the attributes of dining fashion and price. Please choose a restaurant you prefer to visit.

| Attributes | Restaurant A | Restaurant B |
| --- | --- | --- |
| Dining fashion | Top ingredients | Menu innovation |
| Price | Low | High |
| Which restaurant would you choose? | □Restaurant A | □Restaurant B |

1. Two restaurants A and B satisfy all your needs except for the attributes of dining fashion and price. Please choose a restaurant you prefer to visit.

| Attributes | Restaurant A | Restaurant B |
| --- | --- | --- |
| Dining fashion | No top ingredients | Top ingredients |
| Price | Low | High |
| Which restaurant would you choose? | □Restaurant A | □Restaurant B |

1. Two restaurants A and B satisfy all your needs except for the attributes of dining fashion and price. Please choose a restaurant you prefer to visit.

| Attributes | Restaurant A | Restaurant B |
| --- | --- | --- |
| Dining fashion | Media recommendation | Top ingredients |
| Price | Low | High |
| Which restaurant would you choose? | □Restaurant A | □Restaurant B |

1. Two restaurants A and B satisfy all your needs except for the attributes of dining fashion and price. Please choose a restaurant you prefer to visit.

| Attributes | Restaurant A | Restaurant B |
| --- | --- | --- |
| Dining fashion | Menu innovation | Top ingredients |
| Price | Low | High |
| Which restaurant would you choose? | □Restaurant A | □Restaurant B |

1. Two restaurants A and B satisfy all your needs except for the attributes of dining fashion and price. Please choose a restaurant you prefer to visit.

| Attributes | Restaurant A | Restaurant B |
| --- | --- | --- |
| Dining fashion | Menu innovation | Top ingredients |
| Price | Low | High |
| Which restaurant would you choose? | □Restaurant A | □Restaurant B |

1. Two restaurants A and B satisfy all your needs except for the attributes of food, service and price. Please choose a restaurant you prefer to visit.

| Attributes | Restaurant A | Restaurant B |
| --- | --- | --- |
| Food | Average | Good |
| Service | Average | Average |
| Price | Low | High |
| Which restaurant would you choose? | □Restaurant A | □Restaurant B |

1. Two restaurants A and B satisfy all your needs except for the attributes of food, service and price. Please choose a restaurant you prefer to visit.

| Attributes | Restaurant A | Restaurant B |
| --- | --- | --- |
| Food | Average | Good |
| Service | Average | Poor |
| Price | Low | High |
| Which restaurant would you choose? | □Restaurant A | □Restaurant B |

1. Two restaurants A and B satisfy all your needs except for the attributes of food, service and price. Please choose a restaurant you prefer to visit.

| Attributes | Restaurant A | Restaurant B |
| --- | --- | --- |
| Food | Average | Poor |
| Service | Average | Average |
| Price | Low | High |
| Which restaurant would you choose? | □Restaurant A | □Restaurant B |

1. Two restaurants A and B satisfy all your needs except for the attributes of food, service and price. Please choose a restaurant you prefer to visit.

| Attributes | Restaurant A | Restaurant B |
| --- | --- | --- |
| Food | Average | Poor |
| Service | Average | Poor |
| Price | Low | High |
| Which restaurant would you choose? | □Restaurant A | □Restaurant B |

1. Two restaurants A and B satisfy all your needs except for the attributes of food, service and price. Please choose a restaurant you prefer to visit.

| Attributes | Restaurant A | Restaurant B |
| --- | --- | --- |
| Food | Average | Average |
| Service | Average | Good |
| Price | Low | High |
| Which restaurant would you choose? | □Restaurant A | □Restaurant B |

1. Two restaurants A and B satisfy all your needs except for the attributes of food, service and price. Please choose a restaurant you prefer to visit.

| Attributes | Restaurant A | Restaurant B |
| --- | --- | --- |
| Food | Average | Poor |
| Service | Average | Good |
| Price | Low | High |
| Which restaurant would you choose? | □Restaurant A | □Restaurant B |

1. Two restaurants A and B satisfy all your needs except for the attributes of food, service and price. Please choose a restaurant you prefer to visit.

| Attributes | Restaurant A | Restaurant B |
| --- | --- | --- |
| Food | Average | Average |
| Service | Average | Poor |
| Price | Low | High |
| Which restaurant would you choose? | □Restaurant A | □Restaurant B |

1. Two restaurants A and B satisfy all your needs except for the attributes of food, service and price. Please choose a restaurant you prefer to visit.

| Attributes | Restaurant A | Restaurant B |
| --- | --- | --- |
| Food | Average | Good |
| Service | Average | Good |
| Price | Low | High |
| Which restaurant would you choose? | □Restaurant A | □Restaurant B |

1. Two restaurants A and B satisfy all your needs except for the attributes of food, service and price. Please choose a restaurant you prefer to visit.

| Attributes | Restaurant A | Restaurant B |
| --- | --- | --- |
| Food | Average | Good |
| Service | Average | Poor |
| Price | Low | High |
| Which restaurant would you choose? | □Restaurant A | □Restaurant B |

1. Two restaurants A and B satisfy all your needs except for the attributes of food, service and price. Please choose a restaurant you prefer to visit.

| Attributes | Restaurant A | Restaurant B |
| --- | --- | --- |
| Food | Average | Poor |
| Service | Average | Good |
| Price | Low | High |
| Which restaurant would you choose? | □Restaurant A | □Restaurant B |
