# Supplementary material for: Constructing a consumption model of fine dining from the perspective of behavioral economics
Source: PLoS One. 2018 Apr 11;13(4):e0194886. doi: 10.1371/journal.pone.0194886 (PMC5895011; doi:10.1371/journal.pone.0194886)
Supplement: S2 File — (DOCX) [file pone.0194886.s002.docx]

**高價餐廳消費行為之問卷調查**

| 親愛的消費者，您好：  本研究主要探討高價餐廳的消費行為，邀請您填答問卷。這是一份針對消費者對於高價餐廳選擇之行為研究，想瞭解您對選擇此類餐廳用餐的考量因素。此份問卷以不具名，僅作為學術分析，不涉及任何商業利益。感謝您撥冗參與這次研究。  中華大學科技管理博士學程  徐聖訓  蕭振福 敬上 |
| --- |

第一部分 基本資料

1.性別： □男 □女

2.年齡： □20歲以下 □20~29歲 □30~39歲 □40~49歲 □50~59歲
 □60歲(含)以上

3.請問您每週的飲食預算(新台幣)?

□6,000以下 □6,000~11,999 □12,000~17,999 □18,000~23,999
□24,000~29,999 □30,000~35,999 □36,000~41,999 □42,000以上

4.請問您每週花在外出用餐的大概金額(新台幣)?
□3,000以下 □3,000~5,999 □6,000~8,999 □9,000~11,999
□12,000~14,999 □15,000~17,999 □18,000~20,999 □21,000以上

5.請問您每週外出用餐的平均次數?
□0次 □1或2次 □3或4次 □5或6次 □7或8次 □8次以上

第二部分 高價餐廳品質屬性的績效表現與整體滿意度

如果您曾光顧某家高價餐廳，請針對下列五項品質屬性，勾選您感受到的屬性績效表現，並請就這五項屬性的績效表現給予這家餐廳整體滿意度評分。

| **餐廳的品質屬性** | **極不滿意** |  | **不滿意** |  | **略不滿意** | **略滿意** |  | **滿意** |  | **極為滿意** |
| --- | --- | --- | --- | --- | --- | --- | --- | --- | --- | --- |
|  | 1 | 2 | 3 | 4 | 5 | 6 | 7 | 8 | 9 | 10 |
| 1.菜餚與味道(食物) | □ | □ | □ | □ | □ | □ | □ | □ | □ | □ |
| 2.食材安全與料理衛生(食安) | □ | □ | □ | □ | □ | □ | □ | □ | □ | □ |
| 3.滿足您社會交際的動機(用餐動機) | □ | □ | □ | □ | □ | □ | □ | □ | □ | □ |
| 4.餐廳的服務(服務) | □ | □ | □ | □ | □ | □ | □ | □ | □ | □ |
| 5.餐廳的飲食風尚與特色(飲食風尚) | □ | □ | □ | □ | □ | □ | □ | □ | □ | □ |
| ※對於您曾光顧的這一家高價餐廳整體表現的滿意度分數，以0~100分來評分，  您會給 分。 | | | | | | | | | | |

第三部分 情境描述

在假日的晚上，您因某種原因要外出用餐，有兩家在地理位置與交通上相當的高端餐廳可供選擇。而食物、食安、用餐動機、服務、飲食風尚這些因素可能影響您選擇餐廳。

在問卷的第三部分問題中，您對食安、用餐動機、飲食風尚屬性有不同的考量，而這兩家餐廳在食物、服務屬性有不同的績效，價格也有差異。請依照您對食物、食安、用餐動機、服務、飲食風尚等因素的考量，在這兩家餐廳不同的屬性種類與績效狀況下進行餐廳的選擇。

請參考下列餐廳屬性的平均績效描述。

1.食物普通：飽足感剛好、烹飪技巧普通、食材新鮮度尚可、食物味道普通、物有所值。

2.服務普通：服務人員專業尚可信賴、服務人員態度普通、人員適時解決問題。

3.價格適中：價格在您認知的高端餐廳中，定價適中。

第四部分 餐廳選擇

1. 有兩家餐廳A、B除了食安一項不同，其餘項目皆滿足您的需求，請在您選擇的餐廳方框中打勾。

| 屬性 | 餐廳A | 餐廳B |
| --- | --- | --- |
| 食安 | 對食材安全*不*滿意 | 對食材安全滿意 |
| 價格 | 低 | 高 |
| 您會選擇哪家餐廳? | □餐廳A | □餐廳B |

1. 有兩家餐廳A、B除了食安一項不同，其餘項目皆滿足您的需求，請在您選擇的餐廳方框中打勾。

| 屬性 | 餐廳A | 餐廳B |
| --- | --- | --- |
| 食安 | 對料理過程衛生滿意 | 對食材安全滿意 |
| 價格 | 低 | 高 |
| 您會選擇哪家餐廳? | □餐廳A | □餐廳B |

1. 有兩家餐廳A、B除了食安一項不同，其餘項目皆滿足您的需求，請在您選擇的餐廳方框中打勾。

| 屬性 | 餐廳A | 餐廳B |
| --- | --- | --- |
| 食安 | 無過敏物 | 對食材安全滿意 |
| 價格 | 低 | 高 |
| 您會選擇哪家餐廳? | □餐廳A | □餐廳B |

1. 有兩家餐廳A、B除了食安一項不同，其餘項目皆滿足您的需求，請在您選擇的餐廳方框中打勾。

| 屬性 | 餐廳A | 餐廳B |
| --- | --- | --- |
| 食安 | 對料理過程衛生*不*滿意 | 對料理過程衛生滿意 |
| 價格 | 低 | 高 |
| 您會選擇哪家餐廳? | □餐廳A | □餐廳B |

1. 有兩家餐廳A、B除了食安一項不同，其餘項目皆滿足您的需求，請在您選擇的餐廳方框中打勾。

| 屬性 | 餐廳A | 餐廳B |
| --- | --- | --- |
| 食安 | 對食材安全滿意 | 對料理過程衛生滿意 |
| 價格 | 低 | 高 |
| 您會選擇哪家餐廳? | □餐廳A | □餐廳B |

1. 有兩家餐廳A、B除了食安一項不同，其餘項目皆滿足您的需求，請在您選擇的餐廳方框中打勾。

| 屬性 | 餐廳A | 餐廳B |
| --- | --- | --- |
| 食安 | 無過敏物 | 對料理過程衛生滿意 |
| 價格 | 低 | 高 |
| 您會選擇哪家餐廳? | □餐廳A | □餐廳B |

1. 有兩家餐廳A、B除了食安一項不同，其餘項目皆滿足您的需求，請在您選擇的餐廳方框中打勾。

| 屬性 | 餐廳A | 餐廳B |
| --- | --- | --- |
| 食安 | 可能有過敏物 | 無過敏物 |
| 價格 | 低 | 高 |
| 您會選擇哪家餐廳? | □餐廳A | □餐廳B |

1. 有兩家餐廳A、B除了食安一項不同，其餘項目皆滿足您的需求，請在您選擇的餐廳方框中打勾。

| 屬性 | 餐廳A | 餐廳B |
| --- | --- | --- |
| 食安 | 對食材安全滿意 | 無過敏物 |
| 價格 | 低 | 高 |
| 您會選擇哪家餐廳? | □餐廳A | □餐廳B |

1. 兩家餐廳A、B除了食安一項不同，其餘項目皆滿足您的需求，請在您選擇的餐廳方框中打勾。

| 屬性 | 餐廳A | 餐廳B |
| --- | --- | --- |
| 食安 | 對料理過程衛生滿意 | 無過敏物 |
| 價格 | 低 | 高 |
| 您會選擇哪家餐廳? | □餐廳A | □餐廳B |

1. 兩家餐廳A、B除了食安一項不同，其餘項目皆滿足您的需求，請在您選擇的餐廳方框中打勾。

| 屬性 | 餐廳A | 餐廳B |
| --- | --- | --- |
| 食安 | 符合各項食安需求 | *不*符合各項食安需求 |
| 價格 | 高 | 低 |
| 您會選擇哪家餐廳? | □餐廳A | □餐廳B |

1. 有兩家餐廳A、B除了用餐動機一項不同，其餘項目皆滿足您的需求，請在您選擇的餐廳方框中打勾。

| 屬性 | 餐廳A | 餐廳B |
| --- | --- | --- |
| 動機 | *不*適合與親人用餐 | 適合與親人用餐 |
| 價格 | 低 | 高 |
| 您會選擇哪家餐廳? | □餐廳A | □餐廳B |

1. 有兩家餐廳A、B除了用餐動機一項不同，其餘項目皆滿足您的需求，請在您選擇的餐廳方框中打勾。

| 屬性 | 餐廳A | 餐廳B |
| --- | --- | --- |
| 動機 | 適合與情人用餐 | 適合與親人用餐 |
| 價格 | 低 | 高 |
| 您會選擇哪家餐廳? | □餐廳A | □餐廳B |

1. 有兩家餐廳A、B除了用餐動機一項不同，其餘項目皆滿足您的需求，請在您選擇的餐廳方框中打勾。

| 屬性 | 餐廳A | 餐廳B |
| --- | --- | --- |
| 動機 | 適合團體用餐 | 適合與親人用餐 |
| 價格 | 低 | 高 |
| 您會選擇哪家餐廳? | □餐廳A | □餐廳B |

1. 有兩家餐廳A、B除了用餐動機一項不同，其餘項目皆滿足您的需求，請在您選擇的餐廳方框中打勾。

| 屬性 | 餐廳A | 餐廳B |
| --- | --- | --- |
| 動機 | *不*適合與情人用餐 | 適合與情人用餐 |
| 價格 | 低 | 高 |
| 您會選擇哪家餐廳? | □餐廳A | □餐廳B |

1. 有兩家餐廳A、B除了用餐動機一項不同，其餘項目皆滿足您的需求，請在您選擇的餐廳方框中打勾。

| 屬性 | 餐廳A | 餐廳B |
| --- | --- | --- |
| 動機 | 適合與親人用餐 | 適合與情人用餐 |
| 價格 | 低 | 高 |
| 您會選擇哪家餐廳? | □餐廳A | □餐廳B |

1. 有兩家餐廳A、B除了用餐動機一項不同，其餘項目皆滿足您的需求，請在您選擇的餐廳方框中打勾。

| 屬性 | 餐廳A | 餐廳B |
| --- | --- | --- |
| 動機 | 適合團體用餐 | 適合與情人用餐 |
| 價格 | 低 | 高 |
| 您會選擇哪家餐廳? | □餐廳A | □餐廳B |

1. 有兩家餐廳A、B除了用餐動機一項不同，其餘項目皆滿足您的需求，請在您選擇的餐廳方框中打勾。

| 屬性 | 餐廳A | 餐廳B |
| --- | --- | --- |
| 動機 | *不*適合團體用餐 | 適合團體用餐 |
| 價格 | 低 | 高 |
| 您會選擇哪家餐廳? | □餐廳A | □餐廳B |

1. 有兩家餐廳A、B除了用餐動機一項不同，其餘項目皆滿足您的需求，請在您選擇的餐廳方框中打勾。

| 屬性 | 餐廳A | 餐廳B |
| --- | --- | --- |
| 動機 | 適合與親人用餐 | 適合團體用餐 |
| 價格 | 低 | 高 |
| 您會選擇哪家餐廳? | □餐廳A | □餐廳B |

1. 有兩家餐廳A、B除了用餐動機一項不同，其餘項目皆滿足您的需求，請在您選擇的餐廳方框中打勾。

| 屬性 | 餐廳A | 餐廳B |
| --- | --- | --- |
| 動機 | 適合與情人用餐 | 適合團體用餐 |
| 價格 | 低 | 高 |
| 您會選擇哪家餐廳? | □餐廳A | □餐廳B |

1. 有兩家餐廳A、B除了用餐動機一項不同，其餘項目皆滿足您的需求，請在您選擇的餐廳方框中打勾。

| 屬性 | 餐廳A | 餐廳B |
| --- | --- | --- |
| 動機 | 符合用餐動機 | *不*符合用餐動機 |
| 價格 | 高 | 低 |
| 您會選擇哪家餐廳? | □餐廳A | □餐廳B |

1. 有兩家餐廳A、B除了飲食風尚一項不同，其餘項目皆滿足您的需求，請在您選擇的餐廳方框中打勾。

| 屬性 | 餐廳A | 餐廳B |
| --- | --- | --- |
| 風尚 | 沒有媒體推薦 | 媒體推薦 |
| 價格 | 低 | 高 |
| 您會選擇哪家餐廳? | □餐廳A | □餐廳B |

1. 有兩家餐廳A、B除了飲食風尚一項不同，其餘項目皆滿足您的需求，請在您選擇的餐廳方框中打勾。

| 屬性 | 餐廳A | 餐廳B |
| --- | --- | --- |
| 風尚 | 講求菜色創新 | 媒體推薦 |
| 價格 | 低 | 高 |
| 您會選擇哪家餐廳? | □餐廳A | □餐廳B |

1. 有兩家餐廳A、B除了飲食風尚一項不同，其餘項目皆滿足您的需求，請在您選擇的餐廳方框中打勾。

| 屬性 | 餐廳A | 餐廳B |
| --- | --- | --- |
| 風尚 | 訴求頂級食材 | 媒體推薦 |
| 價格 | 低 | 高 |
| 您會選擇哪家餐廳? | □餐廳A | □餐廳B |

1. 有兩家餐廳A、B除了飲食風尚一項不同，其餘項目皆滿足您的需求，請在您選擇的餐廳方框中打勾。

| 屬性 | 餐廳A | 餐廳B |
| --- | --- | --- |
| 風尚 | 菜色*不*夠創新 | 講求菜色創新 |
| 價格 | 低 | 高 |
| 您會選擇哪家餐廳? | □餐廳A | □餐廳B |

1. 有兩家餐廳A、B除了飲食風尚一項不同，其餘項目皆滿足您的需求，請在您選擇的餐廳方框中打勾。

| 屬性 | 餐廳A | 餐廳B |
| --- | --- | --- |
| 風尚 | 媒體推薦 | 講求菜色創新 |
| 價格 | 低 | 高 |
| 您會選擇哪家餐廳? | □餐廳A | □餐廳B |

1. 有兩家餐廳A、B除了飲食風尚一項不同，其餘項目皆滿足您的需求，請在您選擇的餐廳方框中打勾。

| 屬性 | 餐廳A | 餐廳B |
| --- | --- | --- |
| 風尚 | 訴求頂級食材 | 講求菜色創新 |
| 價格 | 低 | 高 |
| 您會選擇哪家餐廳? | □餐廳A | □餐廳B |

1. 有兩家餐廳A、B除了飲食風尚一項不同，其餘項目皆滿足您的需求，請在您選擇的餐廳方框中打勾。

| 屬性 | 餐廳A | 餐廳B |
| --- | --- | --- |
| 風尚 | *未*訴求頂級食材 | 訴求頂級食材 |
| 價格 | 低 | 高 |
| 您會選擇哪家餐廳? | □餐廳A | □餐廳B |

1. 有兩家餐廳A、B除了飲食風尚一項不同，其餘項目皆滿足您的需求，請在您選擇的餐廳方框中打勾。

| 屬性 | 餐廳A | 餐廳B |
| --- | --- | --- |
| 風尚 | 媒體推薦 | 訴求頂級食材 |
| 價格 | 低 | 高 |
| 您會選擇哪家餐廳? | □餐廳A | □餐廳B |

1. 有兩家餐廳A、B除了飲食風尚一項不同，其餘項目皆滿足您的需求，請在您選擇的餐廳方框中打勾。

| 屬性 | 餐廳A | 餐廳B |
| --- | --- | --- |
| 風尚 | 講求菜色創新 | 訴求頂級食材 |
| 價格 | 低 | 高 |
| 您會選擇哪家餐廳? | □餐廳A | □餐廳B |

1. 有兩家餐廳A、B除了飲食風尚一項不同，其餘項目皆滿足您的需求，請在您選擇的餐廳方框中打勾。

| 屬性 | 餐廳A | 餐廳B |
| --- | --- | --- |
| 風尚 | 符合飲食風尚需求 | *不*符合飲食風尚需求 |
| 價格 | 高 | 低 |
| 您會選擇哪家餐廳? | □餐廳A | □餐廳B |

1. 有兩家餐廳A、B除了下列情況不同，其餘項目皆滿足您的需求，請在您選擇的餐廳方框中打勾。

| 屬性 | 餐廳A | 餐廳B |
| --- | --- | --- |
| 食物 | 平均 | 優良 |
| 服務 | 平均 | 平均 |
| 價格 | 低 | 高 |
| 您會選擇哪家餐廳? | □餐廳A | □餐廳B |

1. 有兩家餐廳A、B除了下列情況不同，其餘項目皆滿足您的需求，請在您選擇的餐廳方框中打勾。

| 屬性 | 餐廳A | 餐廳B |
| --- | --- | --- |
| 食物 | 平均 | 優良 |
| 服務 | 平均 | 不佳 |
| 價格 | 低 | 高 |
| 您會選擇哪家餐廳? | □餐廳A | □餐廳B |

1. 有兩家餐廳A、B除了下列情況不同，其餘項目皆滿足您的需求，請在您選擇的餐廳方框中打勾。

| 屬性 | 餐廳A | 餐廳B |
| --- | --- | --- |
| 食物 | 平均 | 不佳 |
| 服務 | 平均 | 平均 |
| 價格 | 高 | 低 |
| 您會選擇哪家餐廳? | □餐廳A | □餐廳B |

1. 有兩家餐廳A、B除了下列情況不同，其餘項目皆滿足您的需求，請在您選擇的餐廳方框中打勾。

| 屬性 | 餐廳A | 餐廳B |
| --- | --- | --- |
| 食物 | 平均 | 不佳 |
| 服務 | 平均 | 不佳 |
| 價格 | 高 | 低 |
| 您會選擇哪家餐廳? | □餐廳A | □餐廳B |

1. 有兩家餐廳A、B除了下列情況不同，其餘項目皆滿足您的需求，請在您選擇的餐廳方框中打勾。。

| 屬性 | 餐廳A | 餐廳B |
| --- | --- | --- |
| 食物 | 平均 | 平均 |
| 服務 | 平均 | 優良 |
| 價格 | 低 | 高 |
| 您會選擇哪家餐廳? | □餐廳A | □餐廳B |

1. 有兩家餐廳A、B除了下列情況不同，其餘項目皆滿足您的需求，請在您選擇的餐廳方框中打勾。。

| 屬性 | 餐廳A | 餐廳B |
| --- | --- | --- |
| 食物 | 平均 | 不佳 |
| 服務 | 平均 | 優良 |
| 價格 | 低 | 高 |
| 您會選擇哪家餐廳? | □餐廳A | □餐廳B |

1. 有兩家餐廳A、B除了下列情況不同，其餘項目皆滿足您的需求，請在您選擇的餐廳方框中打勾。

| 屬性 | 餐廳A | 餐廳B |
| --- | --- | --- |
| 食物 | 平均 | 平均 |
| 服務 | 平均 | 不佳 |
| 價格 | 高 | 低 |
| 您會選擇哪家餐廳? | □餐廳A | □餐廳B |

1. 兩家餐廳A、B除了下列情況不同，其餘項目皆滿足您的需求，請在您選擇的餐廳方框中打勾。

| 屬性 | 餐廳A | 餐廳B |
| --- | --- | --- |
| 食物 | 平均 | 優良 |
| 服務 | 平均 | 優良 |
| 價格 | 低 | 高 |
| 您會選擇哪家餐廳? | □餐廳A | □餐廳B |

1. 兩家餐廳A、B除了下列情況不同，其餘項目皆滿足您的需求，請在您選擇的餐廳方框中打勾。

| 屬性 | 餐廳A | 餐廳B |
| --- | --- | --- |
| 食物 | 平均 | 優良 |
| 服務 | 平均 | 不佳 |
| 價格 | 高 | 低 |
| 您會選擇哪家餐廳? | □餐廳A | □餐廳B |

1. 兩家餐廳A、B除了下列情況不同，其餘項目皆滿足您的需求，請在您選擇的餐廳方框中打勾。

| 屬性 | 餐廳A | 餐廳B |
| --- | --- | --- |
| 食物 | 平均 | 不佳 |
| 服務 | 平均 | 優良 |
| 價格 | 高 | 低 |
| 您會選擇哪家餐廳? | □餐廳A | □餐廳B |
